# ﻿Revision of the freshwater crabs of the genus *Tehuana* Rodríguez & Smalley in Smalley 1970 (Decapoda, Pseudothelphusidae), with the descriptions of two new species

**DOI:** 10.3897/zookeys.1117.85362

**Published:** 2022-08-11

**Authors:** Eric G. Moreno-Juárez, José Luis Villalobos, Fernando Álvarez

**Affiliations:** 1 Colección Nacional de Crustáceos, Instituto de Biología, Universidad Nacional Autónoma de México, 04510, Mexico city, Mexico Universidad Nacional Autónoma de México Mexico city Mexico

**Keywords:** Molecular phylogeny, Neotropical region, southeastern Mexico, *Tehuanaayotzintepecensis* sp. nov., *Tehuanacol* sp. nov.

## Abstract

The freshwater crab genus *Tehuana* Rodríguez & Smalley in Smalley, 1970 includes eight species distributed in southeastern Mexico. A recent review of organisms belonging to this genus uncovered new variations in the male gonopod morphology. A phylogenetic analysis based on molecular characters using three genes (H3, 16S, and COI) resulted in the identification of two new species which are described herein: *Tehuanaayotzintepecensis***sp. nov.** from Oaxaca and *Tehuanacol***sp. nov.** from Veracruz. New diagnoses are provided for those species that had very brief descriptions lacking the treatment of important taxonomic characters and an identification key for all the species in the genus is also given. A discussion of the distribution of all the species in *Tehuana* in the Isthmus of Tehuantepec is presented.

## ﻿Introduction

**Figure 1.**
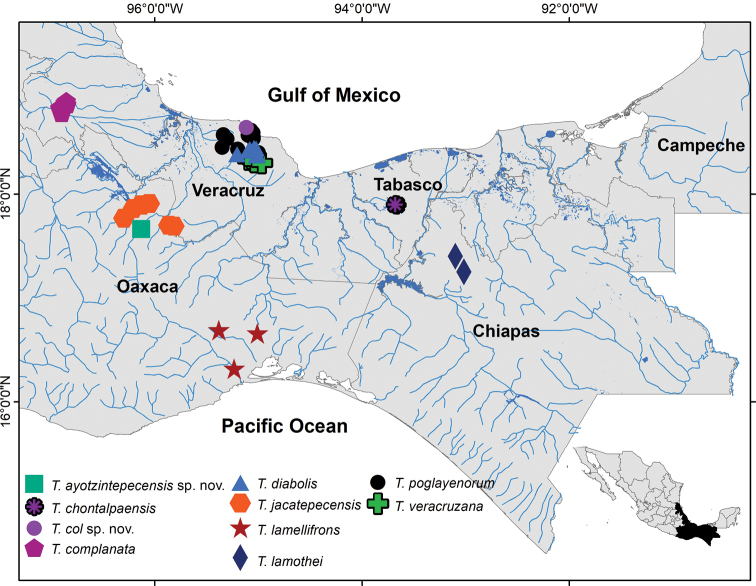
Distribution of the species of the genus *Tehuana* in southeastern Mexico.

The genus *Tehuana* Rodríguez & Smalley in Smalley, 1970, comprises eight species of freshwater crabs of the family Pseudothelphusidae, which are distributed throughout the oriental slope of the states of Veracruz, Oaxaca, and Chiapas, Mexico (Fig. 1). They can be distinguished from other members of the subfamily Pseudothelphusinae (sensu Álvarez et al. 2020) by the morphology of the male first gonopod (G1) which is characterized by an evident meso-distal conical prominence, a strong semicircular carina on the internal surface of the proximal lobe of the caudo-marginal projection and a medial constriction on the lateral surface of the gonopod’s principal axis (Villalobos and Álvarez 2010).

Rodríguez and Smalley (in Smalley 1970) were the first authors to recognize a different and discrete group of species inside the genus *Pseudothelphusa* de Saussure, 1857 with a characteristic morphology of the male G1, and erected the new subgenus Pseudothelphusa (Tehuana) to accommodate three species: Pseudothelphusa (T.) cordobensis Rodríguez & Smalley, 1972, P. (T.) veracruzana Rodríguez & Smalley in Smalley, 1970, and P. (T.) lamellifrons (Rathbun, 1893). Pretzmann (1972) also recognized *Tehuana* as a subgenus of *Pseudothelphusa* including in his monograph: Pseudothelphusa (T.) lamellifrons lamellifrons (Rathbun, 1893), P. (T.) lamellifrons gruneri Pretzmann, 1972 and P. (T.) veracruzana.Pretzmann (1978) subsequently presented two more new subspecies, Pseudothelphusa (T.) lamellifrons poglayenorum and P. (T.) lamellifrons diabolis, from Los Tuxtlas region, Veracruz, Mexico. Later, Pretzmann (1980) presented the same two subspecies with an extended diagnosis. Türkay (1978) revised the nomenclatural status of *T.complanata* (Rathbun, 1905) (= *Pseudothelphusacomplanata* Rathbun, 1905), described for first time the male G1 of the holotype deposited in the Natural History Museum of Paris, and concluded that it belonged in the subgenus Pseudothelphusa (Tehuana).

Rodriguez (1982), in his revision of the freshwater crabs of America, raised P. (Tehuana) to the genus level, and considered that the new genus was closely related to *Pseudothelphusa* and *Epithelphusa* Rodríguez & Smalley in Smalley, 1970 (= *Ehecatusa* Ng & Low, 2010). Furthermore, he placed three species in *Tehuana* and synonymized *T.cordobensis* to *T.complanata*. The two subspecies described by Pretzmann (1980), are mentioned in the Addendum to the monograph without any further analysis, but it is assumed that at that point *Tehuana* included five species.

Alvarez and Villalobos (1994) assigned *Pseudothelphusaguerreroensis* (Rathbun, 1933) to *Tehuana*, although the authors later reassigned this species back to *Pseudothelphusa* based on a morphological phylogeny (Villalobos and Álvarez 2010), and described *T.lamothei* Alvarez & Villalobos, 1994 from Chiapas, Mexico. Villalobos and Alvarez (2003) described *T.chontalpaensis* and *T.jacatepecensis*, from the states of Tabasco and Oaxaca, Mexico, respectively. Villalobos and Alvarez (2010) presented a phylogeny of the tribe Pseudothelphusini (= subfamily Pseudothelphusinae) confirming the close relationship of *Pseudothelphusa* and *Tehuana*. Álvarez et al. (2020) in their revision of the superfamily Pseudothelphusoidea recovered *Tehuana* in the subfamily Pseudothelphusinae, however it appears more closely related to *Disparithelphusa* Smalley & Adkison, 1984 than to *Pseudothelphusa*, as was always considered.

We present a revision of all the material of *Tehuana* deposited in the Colección Nacional de Crustáceos (CNCR) of the Instituto de Biología, Universidad Nacional Autónoma de México (UNAM). We present a revised diagnosis of the genus, the description of two new species, updated diagnoses for four species that lacked sufficient detail in the original descriptions, and an identification key for the species of *Tehuana*. All descriptions and illustrations correspond to the left first male gonopod (G1). In addition, a phylogeny for *Tehuana* and closely related genera based on partial sequences of three genes, two mitochondrial (16S and COI) and one nuclear (H3), is presented. We discuss the relationships among several related genera distributed in southern Mexico and northern Central America.

## ﻿Materials and methods

### ﻿Taxon sampling and morphological characters

**Figure 2.**
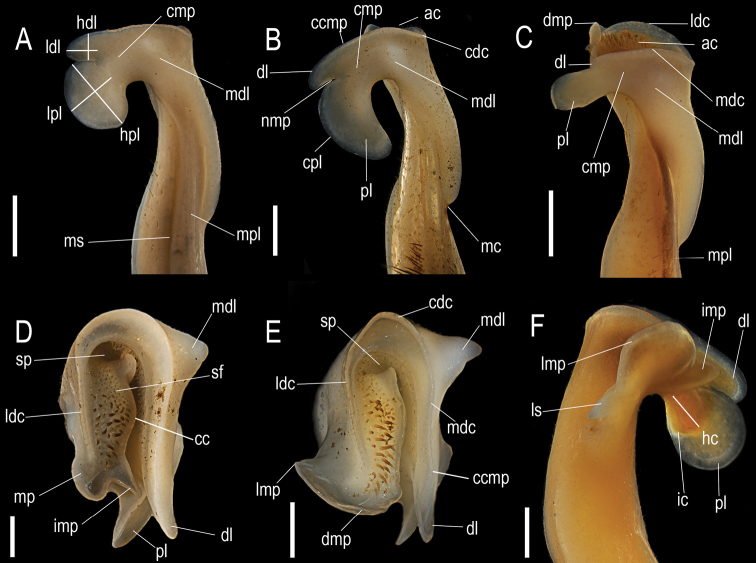
Schematic representation of the terminology used to describe the male first gonopod (G1) of some *Tehuana* species using as examples the G1 of **A***T.lamellifrons***B***T.veracruzana***C***T.lamothei***D***T.jacatepecensis***E***T.poglayenorum***F***T.chontalpaensis.***A–C** mesial view **D, E** distal view **F** lateral view. Scale bars: 1 mm **(A, B, C, F)**; 0.5 mm **(D, E)**. Abbreviations: ac, apical cavity; cc, central crest; cmp, caudo-marginal projection; ccmp, distal crest of the caudo-marginal projection; cdc, caudal distal crest; cpl, cephalic border of the proximal lobe; dl, distal lobe; dmp, distal border of the mesial process; hc, internal carena height; hdl, distal lobe height; hpl, proximal lobe height; ic, internal carena of the proximal lobe; imp, internal angle of the mesial process; ldc, lateral distal crest; ldl, distal lobe length; lmp, lateral border of the mesial process; lpl, proximal lobe length; ls, lateral spine; mc, medial constriction; mdc, mesial distal crest; mdl, meso-distal lobe; mp, mesial process; mpl, marginal plate; ms, marginal suture; nmp, notch of the caudo-marginal projection; pl, proximal lobe; sf, spine field; sp, spermatic pore.

A total of 18 crabs belonging to the genus *Tehuana* was studied. All the specimens are deposited in the Colección Nacional de Crustáceos (**CNCR**) of the Instituto de Biología, Universidad Nacional Autónoma de México (Table 1). The terminology used to describe the male G1 follows Villalobos and Alvarez (2010) (Fig. 2). Photographs of the G1 were taken with a Leica DFC490 camera mounted on a Leica Z16 APOA microscope.

**Table 1.** Specimens used for the phylogenetic analysis of the genus *Tehuana* including taxon name, catalog number in the Colección Nacional de Crustáceos (CNCR), locality, sequenced genes, and GenBank accession numbers.

Species	CNCR	Locality	COI	16S	H3
Subfamily Pseudothelphusinae					
*Tehuanapoglayenorum* (Pretzman, 1978)	33931	Río Basura, San Andrés Tuxtla, Veracruz 18°31'55"N, 95°03'30"W	OK165442	OK256890	OK188918
*Tehuanadiabolis* (Pretzman, 1978)	34488	Río Las Margaritas, Catemaco, Veracruz 18°22'06"N, 95°01'00"W	OK165444	OK256892	OK188920
*Tehuanaveracruzana* Rodríguez & Smalley, in Smalley 1970	33932	Terracería, Zapoapan de Cabañas, Veracruz 18°20'32"N, 95°04'13"W	OK165443	OK256891	OK188919
*Tehuanacol* sp. nov.	33928	Río Col, San Andrés Tuxtla, Veracruz 18°38'29"N, 95°09'00"W	OK165445	OK256893	OK188921
*Tehuanalamellifrons* (Rathbun, 1893)	33939	Nizanda, Asunción Ixtaltepec, Oaxaca 16°41'24” N, 95°22'53” W	OK165446	OK256894	OK188922
*Tehuanacomplanata* (Rathbun, 1905)	11957	Amatlán de Los Reyes, Córdoba, Veracruz 18°51'23"N, 96°54'19"W	OK165447	OK256896	OK188924
*Tehuanaayotzintepecensis* sp. nov.	34628	Arroyo tributario, Río Cajone, Ayotzintepec, Oaxaca 17°39'46"N, 96°07'51"W	OK165448	OK256895	OK188923
*Tehuanajacatepecensis* Villalobos & Alvarez, 2003	11920	Río Santo Domingo, Santa María Jacatepec, Oaxaca 17°51'37"N, 96°12'36"W	–	OK256897	OK188925
*Tehuanachontalpaensis* Villalobos & Alvarez, 2003	25445	Arroyo Frio, Cerro Cola de Sapo; Reserva de la Biósfera Selva El Ocote, Ocozocoautla	MT852948	MT871970	MT860380
*Tehuanalamothei* Alvarez & Villalobos, 1994	8812	Arroyo cerca de Tapilula, Tapilula, Chiapas 18°16'05"N, 93°01'33"W	OK165449	OK256898	OK188926
*Pseudothelphusaamericana* de Saussure, 1857	25527	Río Ajajalpa, Zacatlán, Puebla 19°52'19"N, 97°58'52"W	MT852944	MT871966	MT860376
*Pseudothelphusadoenitzi* Bott, 1968	26190	La Lobera, Zaachila, Oaxaca 16°56'55"N, 96°50'10"W	OK165451	OK256900	OK188928
*Pseudothelphusabelliana* Rathbun, 1898	19228	Chautipan, Chilpancingo, Guerrero 17°30'28"N, 99°44'30"W	MT860377	MT871967	MT852945
*Ehecatusamixtepensis* (Rodríguez & Smalley, 1972)	309	San Gabriel Mixtepec, Oaxaca 16°05'33"N, 97°04'53"W	MT852943	–	MT860375
*Smalleyustricristatus* Alvarez, 1989	7034	Sierra de Santa Marta, Los Tuxtlas, Veracruz 18°26'00"N, 94°57'00"W	MT852947	MT871969	MT860379
*Disparithelphusapecki* Smalley & Adkinson, 1984	34625	Cerro Cangrejo, San Juan Bautista Valle Nacional, Oaxaca 17°48'04"N, 96°19'06"W	OK165450	OK256899	OK188927
Subfamily Raddausinae					
*Odontothelphusalacandona* Alvarez & Villalobos, 1998	11204	Ocosingo, Chiapas 16°25'00"N, 90°30'00” W	MT852048	MT871956	MT860366
*Odontothelphusatoninae* Alvarez & Villalobos, 1991	5770	Ruinas de Toniná, Chiapas 16°54'08"N, 92°00'33"W	MT852049	MT871957	MT860367

Updated diagnoses are provided for *Tehuanadiabolis* (Pretzmann, 1978), *T.lamellifrons* (Rathbun, 1893) and *T.poglayenorum* (Pretzmann, 1978), whose original descriptions are too short and do not include relevant taxonomic characters (Fig. 2A). Abbreviations used are **CL** for carapace length, **CW** for carapace width, and **G1** for the male first gonopod.

### ﻿DNA extraction, amplification, and sequencing

Genomic DNA was extracted from the gill tissue and muscle of pereopods of males preserved in 70–80% ethanol. Extraction was performed with the Animal and fungi DNA preparation kit from Jena Bioscience, following the manufacturer’s protocol. Concentration of DNA was measured with a NanoDrop 2000 spectrophotometer, and the integrity assessed using an agarose gel (1%). Three genes were partially sequenced, two mitochondrial: COX 1 (ChelF1 5’-TAC TCT ACT AAT CAT AAA GAC ATT GG-3’; ChelR1 5’-CCT CCT CCT GAA GGG TCA AAA AAT GA-3’; Barret and Hebert 2005) and 16S (16Sa 5’- ACT TGA TAT ATA ATT AAA GGG CCG-3’; 16Sb (5’-CTG GCG CCG CTC TGA ACT CAA ATC-3’; Palumbi and Benzie 1991); and one nuclear: H3 (H3AF 5’- ATG GCT CGT ACC AAG CAG ACV GC-3, H3AR 5’- ATA TCC TTR GGC ATR ATR GTG AC-3’; Colgan et al. 1998). The polymerase chain reaction (PCR) was performed with MyTaq Kit from Bioline; following the manufacturer’s protocol. The PCR thermic profiles were as follows: COX 1 and H3, with an initial step of 5 min at 95 °C; 35 cycles of 45 s of 94 °C, 45 s at 50 °C and 1 min at 72 °C; with a final extension of 10 min at 72 °C. For the 16S gene: an initial step of 5 min at 95 °C; 35 cycles of 45 s of 94 °C, 45 s at 48 °C and 1 min at 72 °C; with a final extension of 10 min at 72 °C. PCR products were visualized on agarose gel (1%). The sequencing of samples was performed with the reaction kit ABI Prism 3100 Genetic Analyzer, Applied Biosystems automated sequencer.

### ﻿Phylogenetic analysis

The consensus sequences were manually obtained using MEGA v. 7.0 (Kumar et al. 2016) and Finch T.V. v. 1.4.0 (Geospiza Inc.). The presence of stop codons was reviewed with MESQUITE v. 3.6.1 (Maddison and Maddison 2019). Finally, the sequences were compared with online libraries of BLAST and deposited on GenBank (Table 1). The alignment was performed in MAFFT v. 7 (Katoh et al. 2019) with default parameters. Partition for protein sequences genes were performed as in Álvarez et al. (2020), one partition for each gene. The best fit model was obtained in jModelTest v. 2.1.10 (Darriba et al. 2012), with the Akaike Information Criterion (**AIC**) (Hurvich and Tsai 1989). The final concatenated matrix of 1,368 base pairs was analyzed with two phylogenetic inference methods, maximum likelihood (**ML**) and Bayesian inference (**BI**). The maximum likelihood tree was performed in RAxML-HPC BlackBox v.8.2.12 (Stamatakis 2014) in CIPRES (Miller et al. 2010). The optimal number of pseudoreplicates was calculated by the program and the tree with the best bootstrap values was chosen. We only presented the concatenated tree and report clades with branch support above 50%. The Bayesian inference analysis was run in MrBayes v. 3.2.7 (Ronquist and Huelsenbeck 2003) in CIPRES, with the previously inferred substitution models. The parameters were as follows: two independent runs with four Monte Carlo Markov chains, a temperature of 0.1, 10,000,000 generations sampling every 1,000 generations, and a burn-in of 25%. A majority consensus tree was obtained, only clades with a branch support greater than 50% are reported. The convergence of the chains and the optimal ESS values were corroborated in Tracer v. 1.7.1 (Rambaut et al. 2018), as well as the optimal PSRF values were corroborated at the end of the analysis (Gelman and Rubin 1992). Both topologies were visualized in Figtree v. .4.3 (Rambaut et al. 2018).

## ﻿Results

### ﻿Phylogenetic analysis

For the ML analysis in RAxML-HPC BlackBox v. 8.2.12, each gene was analyzed under the GTR model (Tavaré 1986), assuming the following parameters: COI nucleotide frequencies: A = 0.3236; C = 0.1771; G = 0.1498; T = 0.3493; substitution model: A/C: 1.34; A/G: 4.84; A/T: 2.34; C/G: 0.33; C/T: 20.33: G/T: 1.00. 16S nucleotide frequencies: A = 0.3581; C = 0.0990; G = 0.1787; T = 0.3640; substitution model: A/C: 0.65; A/G: 15.21; A/T: 2.30; C/G: 0.00; C/T: 4.32; G/T: 1.00. H3 nucleotide frequencies: A = 0.2065; C = 0.3160; G = 0.2787; T = 0.1986; substitution model: A/C: 0.02; A/G: 0.11; A/T: 0.02; C/G: 0.00; C/T: 0.15; G/T: 1.00. For the BI analysis in MrBayes v. 3.2.7 the following models were assumed COI: GTR + G with a gamma distribution of 0.15 and nucleotide frequencies of A = 0.3157, C = 0.1729, G = 0.1550, 0.3564; 16S, GTR + I with an invariable site frequency of 0.6780 and nucleotide frequencies of A = 0.3729, C = 0.0935, G = 0.1689, T = 0.3647; and H3, F81 (Felsenstein 1981), with nucleotide frequencies of A = 0.2076, C = 0.3126, G = 0.2723, T = 0.2075.

**Figure 3.**
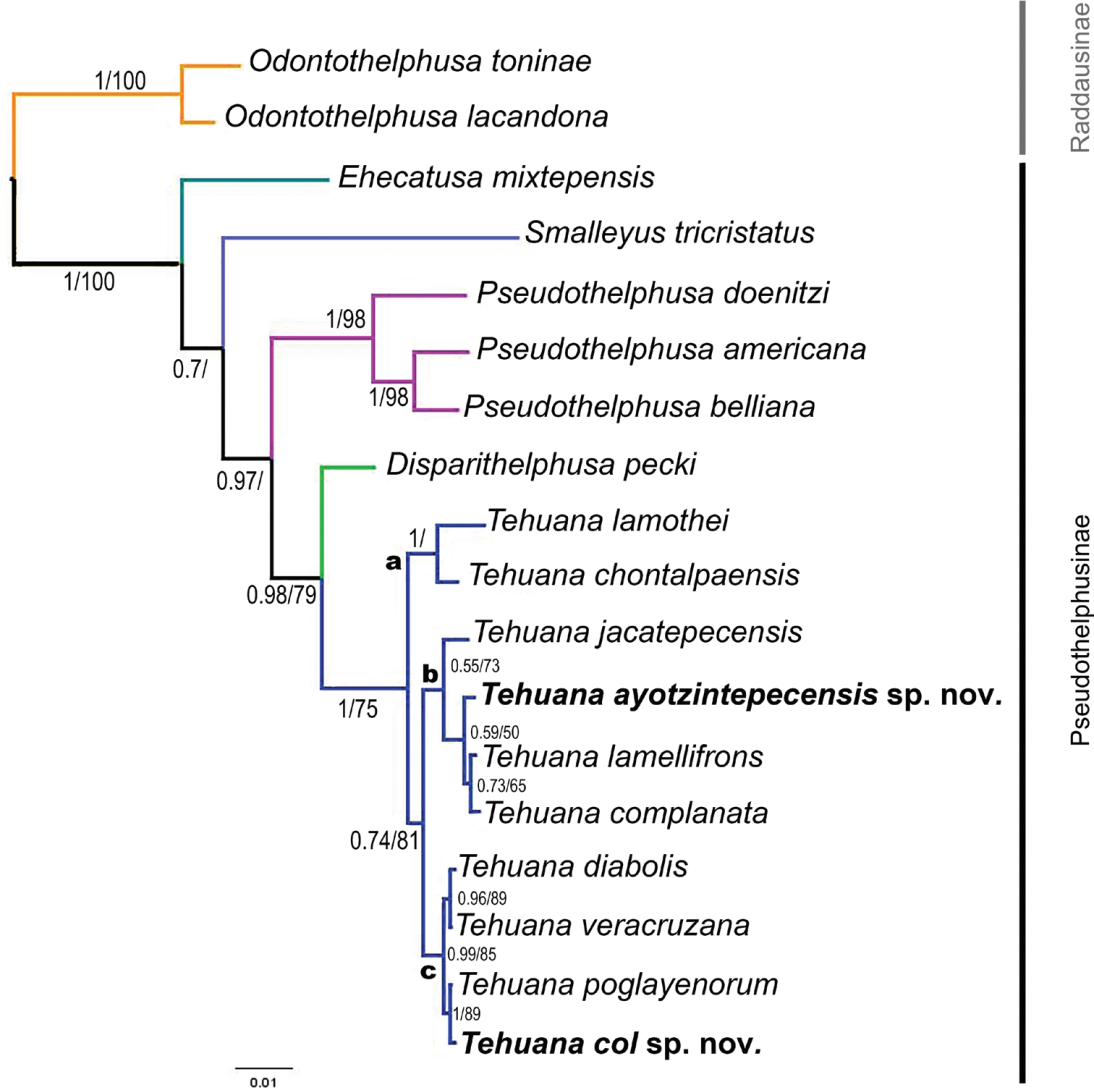
Phylogenetic tree obtained for the genus *Tehuana* based on the concatenated analysis of three genes (H3, 16S, COI), using maximum likelihood and Bayesian inference. Branch supports values are posterior probability/bootstrap. Only clades with branch support above 50% indicated. Clades a, b, and c are labelled for discussion purposes.

The phylogenetic analysis resolved the genus *Tehuana* as monophyletic with an internal organization of three main clades (Fig. 3, clades a, b, c), which are consistent with the male G1 morphology. Species in clade a have a well-developed laminar mesial process in longitudinal position, laterally or caudally oriented with a wide distal border and a strong and acute lateral median spine. Species of clade b have a transversal, reduced, cylindrical with the distal surface of mesial process excavated, without lateral ornamentation and with the internal angle developed as a triangular projection. Species in clade c have a moderately developed mesial process longitudinal, oriented laterally, ornamented with a small lateral median spine.

The arrangement of the species of *Tehuana* within the tree is also concordant with their geographical distribution: clade a, *Tehuanalamothei* and *T.chontalpaensis* from Chiapas and Tabasco, respectively; clade b, *T.jacatepecensis*, *T.ayotzintepecensis* sp. nov., *T.lamellifrons*, and *T.complanata*, from the southwestern margins of the Papaloapan River basin along the Sierra Norte, Oaxaca; and clade c, *T.diabolis*, *T.veracruzana*, *T.poglayenorum*, and *T.col* sp. nov., from Los Tuxtlas region, Veracruz (Figs 1, 3).

### ﻿Taxonomy

**Family Pseudothelphusidae Ortmann, 1893**

#### Subfamily Pseudothelphusinae Ortmann, 1893

##### ﻿Genus Tehuana

Taxon classificationAnimaliaAsteralesAsteraceae

Rodríguez & Smalley in Smalley, 1970

AA0A15F3-CFCC-5A6E-A126-DB2DBB6231EF

Pseudothelphusa (Tehuana) Rodríguez & Smalley in Smalley, 1970: 106 (in key).—Pretzmann 1971: 22.—Pretzmann 1972: 107; 1980: 660.—Rodríguez and Smalley 1972: 77.—Türkay 1978: 144.
Tehuana .—Rodriguez, 1982: 129; 1986: 66.—Villalobos-Hiriart et al. 1993: 285 (list).—Alvarez and Villalobos 1994: 730.—Alvarez et al. 1996: 129.—Villalobos and Alvarez 2003: 228.—Álvarez et al. 2005: 191.—Ng et al. 2008: 177 (list).—Villalobos Hiriart and Álvarez 2008: 279.—Villalobos and Alvarez 2010: 474.—Álvarez et al. 2011b: 289.—Mejía-Ortíz et al. 2011: 19 (list).—Guinot and Hendrickx 2014: 477, 478, tab. 1.—Alvarez and Villalobos 2016: 244.—Villalobos-Hiriart et al. 2019: 156, tab.1 (list).—Álvarez et al. 2020: 12, tab. 4, 20 (list).

###### Type species.

*Pseudothelphusalamellifrons* Rathbun, 1893 [by original designation].

###### Diagnosis.

Carapace with dorsal surface flat, smooth, punctate, with small granulations adjacent to antero-lateral margin. Front vertically deflexed, smooth, superior border formed by low rounded tubercles, medially divided by V-shaped notch reaching inferior margin; inferior margin not distinguishable dorsally, in ventral view projected, widely bilobed. Third maxillipeds with ischium trapezoidal, slightly longer than wide; merus smaller than ischium, anterolateral margin rounded, with anterior shallow concavity; ratio exopod/ischium length 0.6 to 0.8. G1 slender, proximal half cylindrical, compressed distally, with a meso-distal lobe conical, well developed on mesial surface; principal axis with medial constriction on lateral surface and twisted towards medial suture of ventral sternites (Fig. 2). In mesial view, proximal half with marginal plate closing spermatic channel, border facing marginal suture with proximal fringe of long and stout setae; distal half inclined towards cephalic surface; caudal surface fused with marginal plate and expanded cephalically to form the bilobed caudo-marginal projection, distal lobe well-developed (except in *T.lamothei* where it is reduced), subrectangular with cephalic border rounded, distal portion can be curved proximally and separated from proximal lobe by an incision; proximal lobe well developed or reduced, trapezoidal, circular, ax-shaped or elongated proximally to reach and sometimes overlap gonopod principal axis (Fig. 2B). Meso-distal lobe arising from distocaudal angle of mesial surface, conical, subacute, or flattened caudo-cephalically, distally rounded. In cephalic view, mesial process in longitudinal position, oval shaped, closing apical cavity, distal margin widely rounded, lateral margin straight, armed with strong median spine (*T.chontalpaensis*, *T.lamothei*), or with moderate or reduced triangular tooth (*T.diabolis*, *T.col* sp. nov., *T.poglayenorum*, *T.veracruzana*); or in transversal position, reduced, spoon shaped, with lateral margin smooth without spine or tooth (*T.ayotzintepecensis* sp. nov., *T.complanata*, *T.jacatepecensis*, *T.lamellifrons*). Caudo-marginal projection with the two lobes directed cephalically, distal one slightly curved mesially; proximal one, ax-shaped or elongated proximally, and inclined in different gradations; carina of inner surface partially visible. Meso-distal lobe arise from caudal corner of G1 mesial surface, commonly is well developed, conical, with subacute or rounded apex. In lateral view, distal half inclined cephalically. Caudo-marginal projection with distal lobe (partially or totally visible; inner surface of proximal lobe with circular or semicircular strong carina, extending to different extents over proximal surface. In caudal view, distal third straight, apical cavity distally directed, caudal surface ending distally in a wide and shallow concavity. Caudo-marginal projection with the distal crest of mesial surface higher or at the same level as lateral. Meso-distal lobe well developed, conical, with the apex rounded or subacute rounded, its position with respect the distal crest of mesial surface, could variate through different species. Mesial process as a longitudinal apical plate or transversal reduced with the distal surface excavated, in the first case, oval shaped, the distal margin could be widely rounded, and the lateral margin straight and armed with a strong median spine, or with a moderate or reduced triangular tooth; in the second, only partially visible. In distal view, apical cavity U-shaped, opening cephalically. Field of apical setae with 20 to 60 setae; aperture of spermatic channel in caudal position; central crest ending cephalically in acute and triangular or rounded internal angle of mesial process (Fig. 2D, E).

###### Remarks.

The phylogeny presented shows *Tehuana* to be closely related to *Disparithelphusa* (Fig. 2); however, there are strong differences in the G1 morphology: in *Tehuana* it is stouter, the lateral notch on the main shaft is in the middle, the internal surface of the proximal lobe of the caudo-marginal projection is marked with a strong carina, and the meso-distal lobe arises from the distocaudal corner of the mesial surface. Álvarez et al. (2020) in their phylogenetic analysis of the family also recovered *Tehuana* and *Disparithelphusa* as sister taxa.

###### Distribution.

The species of *Tehuana* are distributed in southeastern Mexico covering the Isthmus of Tehuantepec region (Fig. 1) in Chiapas, Oaxaca, Tabasco, and Veracruz.

###### Species included.

*Tehuanaayotzintepecensis* sp. nov.; *T.col* sp. nov.; *T.complanata* (Rathbun, 1905); *T.chontalpaensis* Villalobos & Alvarez, 2003; *T.diabolis* (Pretzmann, 1978); *T.jacatepecensis* Villalobos & Alvarez, 2003; *T.lamellifrons* (Rathbun, 1893); *T.lamothei* Alvarez & Villalobos, 1994; *T.poglayenorum* (Pretzmann, 1978); *Tehuanaveracruzana* (Rodríguez & Smalley in Smalley, 1970).

### ﻿Key to the species of *Tehuana* based on the G1 morphology

**Table:** 

1	Mesial process well developed with lateral spine (Fig. 9A)	**5**
–	Mesial process reduced without lateral spine (Fig. 9B)	**2**
2	Mesial process cephalad oriented (Fig. 9H)	***T.jacatepecensis***
–	Mesial process distally oriented (Fig. 9F)	**3**
3	Proximal lobe of CMP with rounded margins (Fig. 8F)	***T.lamellifrons***
–	Proximal lobe of caudo-marginal projection with internal margin straight (Fig. 8A, D)	**4**
4	Distal lobe of CMP as long as proximal one (Fig. 8A)	***T.ayotzintepecensis* sp. nov.**
–	Distal lobe of CMP shorter than proximal one (Fig. 8D	***T.complanata***
5	Lateral spine on lateral border of mesial process large, in proximal third (Fig. 9B, G)	**6**
–	Lateral spine on lateral border of mesial process small or incipient in distal half (Fig. 9C, I, J)	**7**
6	Mesial process oriented laterally, distal lobe of caudo-marginal projection well developed (Figs 8B, 9B)	***T.chontalpaensis***
–	Mesial process oriented caudally, caudally, distal lobe of caudo-marginal projection reduced (Figs 8G, 9G)	***T.lamothei***
7	Apex of gonopod strongly inclined cephalically, lobes of caudo-marginal projection overlapping (Fig. 8C, I)	**8**
–	Apex of gonopod slightly inclined cephalically, lobes of caudo-marginal projection not overlapping (Fig. 8E, J)	**9**
8	Mesial process as widely rounded plate with a proximal triangular tooth (Fig. 9I)	***T.poglayenorum***
–	Mesial process irregular in shape with a sinuous lateral margin (Fig. 9C)	***T.col* sp. nov.**
9	Proximal lobe of caudo-marginal projection 2.5× as high as wide (Fig. 8J)	***T.veracruzana***
–	Proximal lobe of caudo-marginal projection 1.5× as high as wide (Fig. 8E)	***T.diabolis***

#### ﻿ Tehuana ayotzintepecensis sp. nov.

Taxon classificationAnimaliaAsteralesAsteraceae

42D5DEA7-1FFF-5F3B-A507-B1B90AAC5D61

http://zoobank.org/0B1975C1-09E6-4BFC-87CB-F487DAB0AF87

Figs 4 , 5

##### Type material.

***Holotype***: Mexico – Oaxaca • 1 ♂, CL 42.6 mm, CW 67.3 mm; Municipality of Ayotzintepec, Cajone River, south of Ayotzintepec town, stream tributary of Cajone River; 17°39'46"N, 96°07'51"W; alt. 128 m; 5 Mar. 2018; J.L. Villalobos, I.A. Toledano, E.G. Moreno leg; CNCR 34628. ***Paratype***: 1 ♂, CL 43.4 mm, CW 67.4 mm; same as for holotype; CNCR 36323.

##### Additional material examined.

Mexico – Oaxaca • 2 ♂, CL 26.5–28.2 mm, CW 40.6–42.4 mm; same data as for holotype; CNCR 36324.

##### Description.

**Figure 4.**
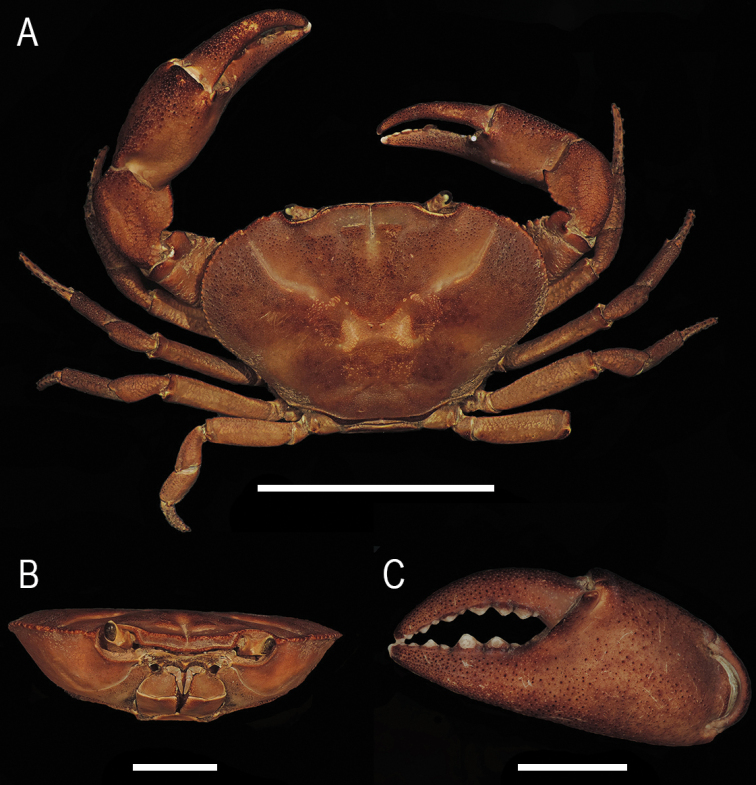
*Tehuanaayotzintepecensis* sp. nov., male holotype **A** total dorsal view **B** frontal view of carapace **C** left chela. Scale bars: 4 cm (**A**); 2 cm (**B, C**).

Carapace with dorsal surface flat, finely punctate, with small black granulations on frontal and anterolateral areas; gastric and branchial regions little inflated; postfrontal portion lightly depressed, almost horizontal, reaching anteriorly superior frontal border. Postfrontal lobes low, but evident, delimited anteriorly by shallow depressions, separated by narrow and deep median groove. Gastric pits deep, wide. Cardiac region discernible. Cervical groove shallow, curved, deep posteriorly, straight anteriorly, becoming obsolete near anterolateral margin, forming shallow notch. Anterolateral margin prominent, armed with 22–24 rounded granules of similar size; granulated between orbit and cervical groove, shallow notch next to orbit. Posterior margin straight (Fig. 4A). In frontal view, superior frontal border straight, formed by low tubercles, divided by deep, V-shaped median notch, external angle internally projected almost touching internal orbital tooth; inferior frontal border thin, granulated, sinuous, more projected than superior one (Fig. 4B). Orbits with external angle slightly granulated, with deep basal notch; internal orbital tooth triangular, well developed, extending to interior of orbit floor as high keel. Basal article of antennal peduncle separated from front by orbital hiatus. Antennules and antennular fossae partially visible, slightly widening in middle portion by an undulation of inferior frontal border; interantennular septum concealed by inferior frontal border. Operculum of antennal gland as ovoidal, flat plate, with middle constriction and tuft of short bristles on lateral third. Epistome devoid of setae; pterygostomian region with low granules; epistomal tooth triangular, directed downwards. Opening of efferent branchial channel subrectangular, longer than wide, width/length ratio 0.68. Third maxilliped with trapezoidal ischium, slightly longer than wide; merus anterior margin rounded with shallow rounded notch in palp articulation; ratio exopod/ischium length 0.70.

Chelipeds distinctly heterochelous in males, subequal size in females. Major chela right, merus subtriangular in cross section, superior margin rounded with short transversal rows of low granules; lower inner margin with longitudinal row of conical tubercles increasing in size distally. Carpus proximal half with row of small conical tubercles, distal with scattered tubercles, ending in short acute spine, median spine obtuse. Fingers moderately gaping, punctate, cutting margins with rounded teeth; fixed finger with row of variable sized subtriangular teeth, median ones larger; tips slightly crossing when closed. Palm slightly swollen (length/width ratio 1.34), inner surface smooth, rest of palm with scattered black granules (Fig. 4C). Dactylus moderately arched, slightly longer than palm (dactylus/palm ratio 1.05).

**Figure 5.**
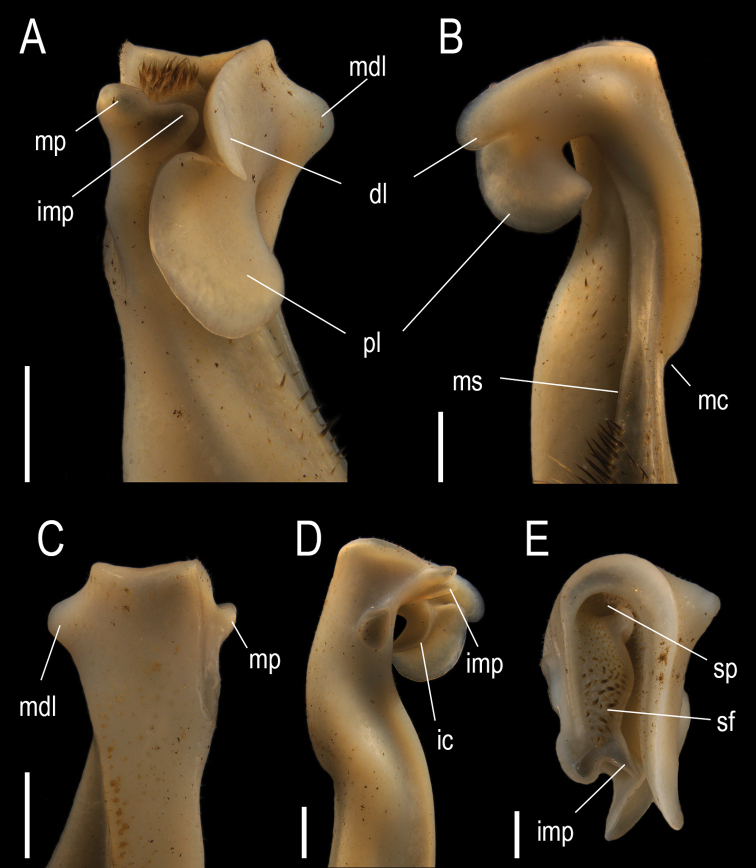
*Tehuanaayotzintepecensis* sp. nov., left G1 of male holotype **A** cephalic view **B** mesial view **C** caudal view **D** lateral view **E** distal view. Abbreviations: dl, distal lobe; ic, interal carena of the proximal lobe; imp, internal angle of the mesial process; ldc, lateral distal crest; mc, middle constriction; mdc, mesial distal crest; mdl, meso-distal lobe; mp, mesial process; ms, marginal suture; pl, proximal lobe; sf, spine field; sp, spermatic pore. Scale bars: 1 mm (**A–D**), 0.5 mm (**E**).

G1 slender, proximal half cylindrical, becoming compressed distally, meso-distal lobe conical, well developed; principal axis with medial constriction on lateral surface, twisted mesially. In mesial view (Fig. 5B), distal half inclined towards cephalic surface. Caudo-marginal projection with distal lobe well developed, subrectangular with cephalic margin rounded, separated from proximal lobe by an incision; proximal lobe well developed, ax-shaped, 1.24 as higher than long. Meso-distal lobe arise from caudal corner of mesial surface, well developed, conical, with rounded apex. In caudal view (Fig. 5C), distal third straight, apical cavity distally directed, caudal surface ending distally in wide, shallow concavity. Caudo-marginal projection distal crest of mesial surface higher than lateral one. Meso-distal lobe well developed, conical, apex rounded. Mesial process reduced, spoon shaped, only partially visible. In cephalic view (Fig. 5A), mesial process reduced, with the distal surface excavated, transversal to principal axis of G1, without spine on lateral margin, laying over carina of inner surface of proximal lobe of caudo-marginal projection; cephalic border bilobed, internal lobe pyramidal, rounded not touching internal face of caudo-marginal projection distal lobe, lateral lobe rounded, subcylindrical, projected anteriorly more than internal one. Field of apical setae visible, cephalo-caudally elongated, setae brownish, shorter than distal crest of lateral surface. Caudo-marginal projection lobes cephalically directed, separated, distal one slightly curved mesially; proximal one oval shaped, lateromesially inclined; carina of inner surface not visible. Distal crest of lateral surface sharp, with some tufts of short setae; subdistal circular scar on lateral face of principal axis partially visible. In lateral view (Fig. 5D), distal half inclined cephalically. Caudo-marginal projection with the distal lobe partially visible, separated from the proximal lobe by incision, as long as lobe; inner surface of proximal lobe with semicircular strong carina, which extends over basal third of proximal surface. Mesial process lateral lobe subcylindrical, superior surface excavated, lateral border smooth, developing proximally rounded margin ending in subcircular scar. In distal view (Fig. 5E), apical cavity U-shaped, opening cephalically. Field of apical setae delimited by central crest and internal surface of lateral surface; 20–60 apical setae; aperture of spermatic channel in caudal position; central crest ending cephalically in acute, triangular internal lobe of mesial process, close to internal surface of distal lobe of caudo-marginal projection. Mesial process with distal surface excavated, as an anterior continuation of the field of setae, raised border delimit the lateral and internal lobes. Meso-distal lobe well developed, conical, apex rounded.

##### Distribution.

Only known from the type locality in northern Oaxaca, Mexico.

##### Etymology.

The specific epithet is taken from the town where the specimens were collected: near town of Ayotzintepec. The word is Náhuatl “Ayotlzin-tepec”, and means “hill of the little turtles” (“en el cerro de las tortuguitas” in Spanish).

##### Remarks.

*Tehuanaayotzintepec* sp. nov. has the typical morphology of the species distributed throughout the Isthmus of Tehuantepec, with a reduced mesial process, cylindrical or spoon shaped, in a transversal position relative to the G1 principal axis and laying over the proximal lobe of the caudo-marginal projection. These characters, make the new species similar to *T.complanata*, *T.jacatepecensis*, and *T.lamellifrons*; however, in *T.ayotzintepecensis* sp. nov. the two lobes of the caudo-marginal projection are of the same length in mesial view, the proximal one has a subacute inner angle and the distal one is completely rounded. Geographically, *T.ayotzintepecensis* sp. nov. and *T.jacatepecensis* occur in the same general area along the Gulf of Mexico versant of the Sierra de Juárez in northern Oaxaca. In contrast, *T.lamellifrons* is distributed along the Pacific versant of the Sierra Madre Occidental in southern Oaxaca, and *T.complanata* occurs in central Veracruz (Fig. 1). The phylogenetic tree is consistent with the morphological similarity as it shows a close relationship between the four species (Fig. 3).

#### ﻿ Tehuana col sp. nov.

Taxon classificationAnimaliaAsteralesAsteraceae

66814882-A723-5BFE-BDFE-AFC3D6AEF630

http://zoobank.org/27434075-2C03-403B-89EE-0A0A412034EE

Figs 6 , 7

##### Type material.

***Holotype***: Mexico – Veracruz • 1 ♂, CL 30.4 mm, CW 50.3 mm; Municipality of San Andrés Tuxtla, Col River at Cascadas Park; 18°38'29"N, 95°09'00"W; alt. 416 m; 25 Apr. 2017; J.L. Villalobos, I.A. Toledano, E.G. Moreno leg; CNCR 33928. Paratype: 1 ♂, CL 13.6 mm, CW 20.8 mm; same data as for holotype; CNCR 36325.

##### Additional material examined.

Mexico – Veracruz • 1 ♀, CL 19.6 mm, CW 29.5 mm; same data as for holotype; CNCR 36325.

##### Description.

**Figure 6.**
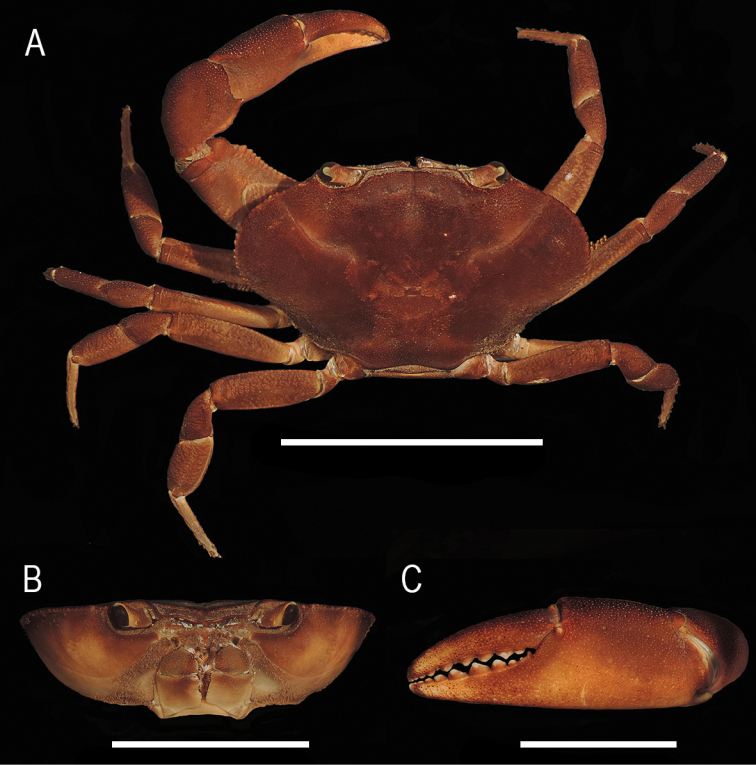
*Tehuanacol* sp. nov., male holotype **A** total dorsal view **B** frontal view of carapace **C** left chela. Scale bars: 4 cm (**A**); 3 cm (**B**); 2 cm (**C**).

Carapace dorsal surface slightly concave, finely punctate, frontal and anterolateral surfaces with minute granulations; gastric and branchial regions slightly inflated; postfrontal portion depressed, almost horizontal, continued anteriorly to reach superior frontal border. Postfrontal lobes low, delimited anteriorly by shallow depressions, separated by narrow, deep median groove. Cardiac region hardly discernible. Cervical groove shallow, curved posteriorly, anterior ¼ straight, becoming obsolete before anterolateral margin, not reaching it. Anterolateral margin prominent, armed with 21–23 conical granules of similar size; portion between orbit and cervical groove granulated, with shallow notch next to orbit. Posterolateral area of carapace with short setae; posterior margin widely concave (Fig. 6A). In frontal view, superior frontal border inclined towards central portion, formed by low tubercles, divided by moderately deep, narrow V-shaped, median notch; inferior frontal border continuous, sinuous, thinner, more projected than superior one (Fig. 6B). Orbits with external angle slightly granulated, with shallow basal notch; internal orbital tooth triangular, well developed, extending to interior of orbit floor as high keel. Basal article of antennal peduncle separated from front by orbital hiatus. Antennules and antennular fossae partially visible, slightly wider in the middle; interantennular septum concealed by inferior frontal border. Operculum of antennal gland as ovoidal, flat plate, with middle constriction, tuft of short bristles on lateral third. Epistome, area around buccal cavity and pterygostomian region with short setae; epistomal tooth covered by patch of short setae, triangular, directed downwards. Opening of branchial efferent channel subcircular, longer than wide, width/length ratio 0.68. Third maxilliped with ischium subrectangular, slightly longer than wide, anterior margin of merus rounded; ratio exopod/ischium length 0.81.

Chelipeds moderately heterochelous in both sexes, more evident in males. Major chela right, merus subtriangular in cross section, superior margin rounded with short transversal rows of low granules; lower inner margin with longitudinal row of conical tubercles increasing in size distally (Fig. 6C).

**Figure 7.**
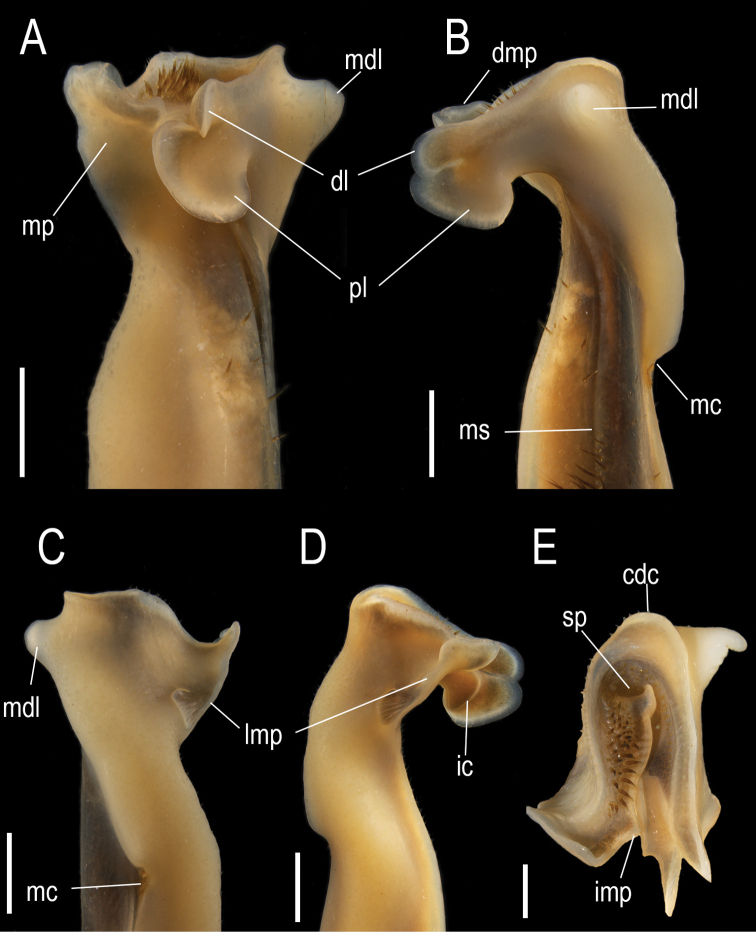
*Tehuanacol* sp. nov., left G1 of male holotype **A** cephalic view **B** mesial view **C** caudal view **D** lateral view **E** distal view. Abbreviations: cdc, caudal distal crest; dl, distal lobe; dmp, distal border of mesial process; ic, internal carena of the proximal lobe; imp, internal angle of the mesial process; mc, middle constriction; mdl, meso-dital lobe; mp, mesial process; ms, marginal suture; pl, proximal lobe; sp, spermatic pore. Scale bars: 1 mm (**A–D**); 0.5 mm (**E**).

G1 slender, proximal half cylindrical, becoming compressed distally, meso-distal lobe on mesial surface conical, well developed; principal axis with medial constriction on lateral surface, twisted mesially. In mesial view (Fig. 7B), distal half inclined towards cephalic surface. Caudo-marginal projection with distal lobe well developed, subrectangular, cephalic margin rounded, separated from proximal lobe by an incision; proximal lobe well developed, ax-shaped, higher than long. Distal crest of MP, partially visible, some rounded and higher than apical cavity. Meso-distal lobe arising from caudal angle of mesial surface, well developed, conical, with subacute apex. In caudal view (Fig. 7C), distal third straight, apical cavity distally directed, caudal surface ending distally in a wide and shallow concavity. Caudo-marginal projection with distal crest of mesial surface as high or higher than lateral one. Mesial process as a longitudinal plate, caudally undulated, partially visible; subdistal circular scar on the base of the plate partially visible. In cephalic view (Fig. 7A), mesial process in longitudinal position relative to principal axis of G1, with an incipient rounded tooth on lateral margin, laying over carina of inner surface of proximal lobe of caudo-marginal projection; cephalic border bilobed, internal lobe pyramidal, rounded not touching internal face of the distal lobe of caudo-marginal projection, lateral lobe rounded, semicylindrical, projected anteriorly beyond internal one. Field of apical setae visible, cephalo-caudally elongated, setae brownish, shorter than distal crest of lateral surface. Lobes of caudo-marginal projection cephalically directed, separated, slightly curved mesially; proximal one oval shaped, lateromesially inclined, inner surface carina not visible. Distal crest of lateral surface sharp, smooth, with few short setae; subdistal circular scar on principal axis partially visible. In lateral view (Fig. 7D), distal lobe of caudo-marginal projection partially visible, separated from proximal one by long incision; inner surface of proximal lobe with circular or semicircular strong carina, extending over basal third of proximal surface. Mesial process as a longitudinal plate that close the apical cavity; lateral margin of smooth, superior angle rounded, median portion with an incipient rounded tooth, continued proximally to end in a subcircular scar. In distal view (Fig. 7E), Apical cavity U-shaped, opening cephalically, field of apical setae delimited by central and lateral crests; 20–60 apical setae; aperture of spermatic channel in caudal position; central crest ending cephalically in acute, triangular internal lobe of mesial process. Meso-distal lobe (MDL) well developed, conical, distal tip curved mesocephalically.

##### Distribution.

Only known from type locality.

##### Etymology.

The name of this species is taken from the River Col, Los Tuxtlas region of Veracruz, where the specimens were collected. We declare the specific epithet as noun in apposition.

##### Remarks.

*Tehuanacol* sp. nov. is morphologically similar to *T.poglayenorum* which occurs in the same area in Los Tuxtlas region, with both exhibiting partially overlapping lobes of the caudo-marginal projection; however, they can be easily separated by the mesial process, irregular shape with a sinuous lateral margin in the former, versus a widely rounded plate with a proximal triangular tooth in the latter. Consistent with the morphology, *T.col* sp. nov. and *T.poglayenorum* are also genetically closely related (Fig. 3), and in turn they are related to *T.diabolis* and *T.veracruzana*. It is interesting to highlight that four clearly defined species of *Tehuana* together with *Smalleyustricristatus* Alvarez, 1989 and *Pseudothelphusaparabelliana* Alvarez, 1989 occur in Los Tuxtlas region which is small mountain range occupying an 80 × 33 km area in the coastal plain of southern Veracruz.

#### ﻿ Tehuana complanata

Taxon classificationAnimaliaAsteralesAsteraceae

(Rathbun, 1905)

27C50A5D-F13E-5283-A541-2709123F12BE

Figs 8D , 9D

? Pseudothelphusa bocourti .—Rathbun, 1898: 512 (in key), 513, 533 (list) [not Bosciabocourti A. Milne-Edwards, 1866].
Pseudothelphusa complanata Rathbun, 1905: 303, fig. 3.—Coifmann 1939: 107 (list).
“Pseudothelphusa” complanata .—Pretzmann, 1965: 10 (list).
Pseudothelphusa (Pseudothelphusa) lamellifronsgruneri.—Pretzmann, 1968: 7.—Pretzmann 1971: 22 (list).
Pseudothelphusa (Pseudothelphusa) americana lamellifrons .—Bott, 1970: 334, pl. 2, figs 11–13.
Potamocarcinus (Raddaus) bocourti complanata .—Pretzmann, 1971: 20.(list).
Potamocarcinus (Raddaus) bocourti complanatus .—Pretzmann, 1972: 78, text fig. 18, fig. 542.
Pseudothelphusa (Tehuana) lamellifrons gruneri.—Pretzmann, 1972: 108, figs 621–623, 674–677.
Pseudothelphusa (Tehuana) cordobensis Rodríguez & Smalley, 1972: 77, fig. 8, pl. 5.
Pseudothelphusa (Tehuana) complanata Türkay, 1978: 145, figs 2a, b, 3.
Tehuana complanata Rodriguez, 1982: 131, fig. 85.—Villalobos 1982: 221 (list).—Villalobos-Hiriart et al. 1993: 284 (list).—Alvarez and Villalobos 1994: 730 .—Álvarez et al. 1999: 20, fig. 3 (map), 23, box 3 (list).—Villalobos and Alvarez 2003: 2003 (in key) .—Villalobos Hiriart and Álvarez 2008: 280, 298 (list).—Ng et al. 2008: 177 (list).—Villalobos and Alvarez 2010: 474, 477, fig. 11 (map).—Álvarez et al. 2011a: appx. VIII.20, p. 13 (list).—Mejía-Ortíz et al. 2011: 97, 136 (map 2).—Álvarez et al. 2012: 1078, box 1 (list).—Cumberlidge et al. 2014: 144, tab. 3 (list).—Alvarez and Villalobos 2016: 254, tab.8.1 (list).—Villalobos-Hiriart et al. 2019: 156, tab.1 (list).

##### Material examined.

Mexico – Veracruz • 1 ♂, holotype of Pseudothelphusa (Tehuana) cordobensisRodríguez and Smalley (1972); LC 38.2 mm, AC 59.0 mm; Municipality of Córdoba Ojo de Agua, Paraje Nuevo, 18°52'35"N, 96°51'49"W; alt. 661 m; 2 May 1953; A. Villalobos leg.; CNCR 311. 1 ♂; LC 30.0 mm, AC 45.4 mm; Municipality of Amatlán de Los Reyes, Lourdes River Cave; 18°47'00"N, 96°54'00"W; alt. 439 m; 13 Jun. 1996; J. Herrera, E. Ramírez leg.; CNCR 11958. 3 ♂, 3 ♀; LC 15.4–27.9 mm, AC 22.9–42.7 mm; Municipality of Amatlán de Los Reyes, Amatlán II Power Station; 18°51'23"N, 96°54'19"W; alt. 730 m; 6 Jun. 1992; J. Herrera, E. Ramírez leg.; CNCR 11957. 1 ♀, LC 26.2 mm, AC 18.9 mm; Motzorongo, Municipality of Tezonapa, Motzorongo River; 18°38'30"N, 96°43'53"W; alt. 271 m; C. Pedraza, L. García leg.; CNCR 34618.

##### Diagnosis.

G1, in cephalic view, with three protuberances on lateral surface, proximal one being the most developed. In mesial view, meso-distal lobe conical, with round and slender apex. In caudal view, median constriction forming a large lobe oriented proximally. Caudo-marginal projection with distal lobes separated by linear notch without leaving space in between. Distal lobe with rounded cephalic edge, shorter than proximal lobe. Proximal lobe ax-shaped, higher than distal (1.5×); cephalic border circular, caudal border straight; distal crest slightly laterally oriented, lobe with sloping appearance. Internal carina well marked, circular, its length covering at least ⅓ of internal surface. Mesial process reduced, in transversal position, without lateral spine; distal edge oriented cephalad. Internal angle developed in form of lobe, two-thirds as high as mesial process, wide, touching the internal surface of distal lobe (DL) of CMP. In distal view, mesial process concave, internal angle hidden below the proximal lobe (PL) of caudo-marginal projection.

##### Distribution.

*Tehuanacomplanata* is distributed around the city of Cordoba, Veracruz, Mexico (Fig. 1).

##### Remarks.

The recognition of the type locality of *Tehuanacomplanata* has been problematic since Rathbun (1905) cited “Coban, Alta Vera Paz, Guatemala” as the type locality of *Pseudothelphusacomplanata.* Later, Rodríguez and Smalley (1972) described Pseudothelphusa (Tehuana) cordobensis from “Paraje Nuevo, Córdoba, Veracruz” which fits the description of *T.complanata*. Türkay (1978) discussed this situation concluding that it was a labelling error by Bocourt who placed crabs from Veracruz, Mexico in a jar with specimens from Coban, Guatemala. Rodriguez (1982) synonymized P. (T.) cordobensis under *T.complanata* and designated the male from “Paraje Nuevo, Córdoba, Veracruz” (CNCR 311) as the holotype.

**Figure 8.**
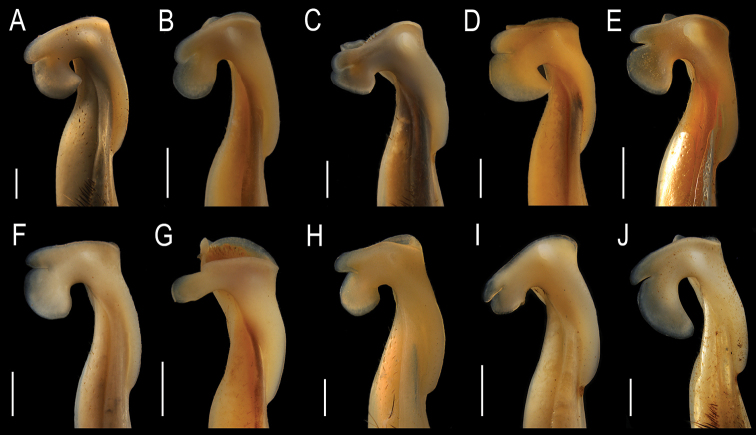
Mesial view of the apical portion of the G1 of the species of *Tehuana***A***T.ayotzintepecensis* sp. nov., CNCR 34628 **B***T.chontalpaensis*, CNCR 17093 **C***T.col* sp. nov., CNCR 33928 **D***T.complanata*, CNCR 11957 **E***T.diabolis*, CNCR 12056 **F***T.lamellifrons*, CNCR 33939 **G***T.lamothei*, CNCR 8812 **H***T.jacatepecensis*, CNCR 11920 **I***T.poglayenorum*, CNCR 33931 **J***T.veracruzana*, CNCR 33934. Scale bars: 1 mm.

Morphologically, the G1 of *T.complanata* is similar to that of *T.jacatepecensis*; both species have a broadly rounded to semicircular proximal lobe of the CMP, although in the former the distal and proximal lobes are subequal in length, whereas in the latter the proximal lobe is clearly shorter (Fig. 8D). *Tehuanacomplanata* is also similar to *T.lamellifrons* even when they are geographically distant within the genus range (Fig. 1); however, in the obtained phylogeny they appear as sister species (Fig. 3).

#### ﻿ Tehuana chontalpaensis

Taxon classificationAnimaliaAsteralesAsteraceae

Villalobos & Alvarez, 2003

17AEDB39-BF00-5BF1-9AB6-F6F54493D1F6

Figs 8B , 9B

Tehuana chontalpaensis Villalobos & Alvarez, 2003: 224, 228 (in key), figs 2, 4A.—Álvarez et al. 2005: 191.—Rodríguez and Magalhães 2005: 356, tab. 1 (list).—Villalobos Hiriart and Álvarez 2008: 280, 298 (list).—Ng et al. 2008: 177 (list).—Villalobos and Alvarez 2010: 474, 477, fig. 11 (map).—Alvarez and Villalobos 2016: 254, tab.8.1 (list).—Villalobos-Hiriart et al. 2019: 156, tab.1 (list).—Álvarez et al. 2020: 979, tab. 1 (list).

##### Material examined.

Mexico – Tabasco • 1 ♂, ***holotype***; LC 35.1 mm, AC 57.3 mm; Municipality of Huimanguillo, Carlos A. Madrazo, Pueblo Viejo Stream; 17°23'45"N, 93°39'45"W; alt. 135 m; 8 May 1997; J.L. Villalobos leg.; CNCR 18952. 2 ♂, paratypes; LC 17.3–24.3 mm, AC 27.7–37.8 mm; same data as for holotype; 12 Jun. 1997; J.L. Villalobos, R. Robles leg.; CNCR 17093. 1 ♂, 1 ♀, paratypes; LC 11.0–14.8 mm, AC 17.0–23.0 mm; same collection data as for holotype; 12 Jun. 1997; J.L. Villalobos, R. Robles leg.; CNCR 17171. 1 ♂, 2 ♀; LC 10.0–22.0 mm, AC 14.2–34.0 mm; Municipality of Huimanguillo, 3 km E of Carlos A. Madrazo, small tributary of Pedregal-Tonala River; 17°23'52"N, 93°40'51"W; alt. 116 m; 22 Jan. 1998; J.L. Villalobos, R. Robles leg.; CNCR 17290. Chiapas 1 ♂; LC 25 mm, AC 42 mm; Municipality of Ocozocoautla, Reserva de la Biósfera Selva el Ocote, Cerro Cola de Sapo, Frio Stream; 18 Dic 2008; A. García and M. Anzueto leg.; CNCR 25445.

##### Diagnosis.

As in Villalobos and Alvarez (2003).

##### Distribution.

Only known from the type locality and surroundings (Fig. 1).

##### Remarks.

As noted by Villalobos and Alvarez (2003) *Tehuanachontalpaensis* is morphologically similar to *T.lamothei*, a similarity that is consistent with their being sister species as shown in the molecular phylogeny (Fig. 3).

#### ﻿ Tehuana diabolis

Taxon classificationAnimaliaAsteralesAsteraceae

(Pretzmann, 1978)

567BB8AE-3493-5B6A-8515-FDDC80EE467B

Figs 8E , 9E

Pseudothelphusa (Tehuana) lamellifrons diabolis Pretzmann, 1978: 3.—Pretzmann 1980: 660, pl. 13, figs 56–60, pl. 17, fig. 77.—Rodriguez 1982: 210.
Tehuana diabolis. —Villalobos-Hiriart et al. 1993: 284 (list) .—Alvarez and Villalobos 1994: 730, 735.—Álvarez and Villalobos 1997a: 416, 417, box 4.17 (list).—Álvarez and Villalobos 1997b: 438, appx. 4.23 (list).—Álvarez et al. 1999: 20, fig. 3 (map), 23, box 3 (list).—Villalobos and Alvarez 2003: 228 (in key).—Villalobos and Álvarez 2008: 281, 298 (list).—Ng et al. 2008: 177 (list).—Villalobos and Alvarez 2010: 474, 477, fig. 11 (map).—Álvarez et al. 2011a: appx. VIII.20, p. 13 (list); 2012: 1078, box 1 (list).—Alvarez and Villalobos 2016: 254, tab. 8.1 (list).—Villalobos-Hiriart et al. 2019: 156, tab.1 (list).—Álvarez et al. 2020: 979, tab. 1 (list).

##### Material examined.

Mexico – Veracruz • 2 ♀, CL 8.2–13.5 mm, CW 12.4–20.2 mm; Municipality of Catemaco, Veracruz, Catemaco Lake, Playa Hermosa; 18°26'00"N, 95°04'60"W; alt. 351 m; 31 Aug. 1966; L. Holthuis, J. Cabrera leg.; CNCR 333. 3 ♂, CL 12.1–16.4 mm, CW 18.1–25.7 mm; Municipality of Catemaco, Catemaco Lake, El Zapotal; 18°25'00"N, 95°05'60"W; alt. 335 m; 18 Sep. 1954; A. Villalobos leg.; CNCR 334. 4 ♂, 6 ♀, CL 12.7–27.1 mm, CW 20.1–42.3 mm; Municipality of Catemaco, Catemaco Lake, Las Margaritas Stream; 18°22'04"N, 95°01'01"W; alt. 345 m; 4 Aug. 1994; M.E. Camacho leg.; CNCR 12956. 4 ♂, CL 12.1–23.4 mm, CW 17.7–38.1 mm; same locality as previous record; 6 Aug. 1994; F. Álvarez leg.; CNCR 12965. 1 ♂, CL 26.4 mm, CW 43.6 mm; same locality as previous record; 20 Apr. 2016; E. Moreno leg.; CNCR 34488. 1 ♂, 1 ♀, CL 12.9–74.6 mm, CW 11.9–44.7 mm; Municipality of Catemaco, 1 km S from Coyame; 18°25'50.6"N, 95°01'16"W; alt. 364 m; 6 Aug. 1994; J.L. Villalobos leg.; CNCR 12952. 2 ♀, CL 11.7–18.2 mm, CW 17.7–23.2 mm; same locality as previous record; 1 Aug. 1994; M.E. Camacho leg.; CNCR 12966. 2 ♀, CL 12.8–15.7 mm, CW 18.8–22.5 mm; Municipality of Catemaco, Catemaco Lake, La Agayota; 18°24'02"N, 95°00'06"W; alt. 545 m; 1 Jul. 1986; F. Álvarez leg.; CNCR 12907. 1 ♂, 4 ♀, CL 11.3–24.6 mm, CW 16.2–38.2 mm; same locality as previous record; 18 Jul. 1986; J.L. Villalobos leg.; CNCR 12911. 1 ♂, CL 13.1 mm, CW 20.1 mm; same locality as previous record; 4 Aug. 1994; F. Álvarez leg.; CNCR 12954. 1 ♀, CL 26.3 mm, CW 42.4 mm; same locality as previous record; 18 Jul. 1986; F. Álvarez leg.; CNCR 13125.

##### Diagnosis.

G1 slender, proximal half cylindrical, distal half becoming compressed. In caudal view, apical crest widely concave, mesial crest higher than lateral one, Meso-distal lobe conical, tip rounded. In mesial view, distal third of gonopod slightly inclined cephalically, caudo-marginal projection bilobed, lobes separated by V-shaped notch, distal lobe shorter than proximal one, rounded; proximal lobe ax-shaped, cephalic margin broadly rounded, internal margin straight. In cephalic view, caudo-marginal projection slightly curved mesially, meso-distal lobe prominent; mesial process as a widely rounded plate, distal margin rounded, lateral margin with triangular tooth, cephalic margin with rounded projection closing the apical cavity. In lateral view, mesial process oblique relative to apical cavity longitudinal axis, mesial crest clearly higher than lateral one. In apical view, apical cavity U-shaped, opening of sperm channel in caudal position, field of apical pore setae on lateral portion of cavity, caudal crest thicker than the rest.

##### Type material.

The holotype (NHMW 4068) and paratypes (NHMW 4069) are deposited in the Natural History Museum in Vienna, Austria.

##### Type locality.

México, Veracruz, Municipality of Catemaco, Catemaco Lake; 18°25'00"N, 95°06'00"W; alt. 325 m (Pretzmann 1980).

##### Distribution.

Only known from the north and eastern shores of Catemaco Lake, Veracruz, Mexico (Fig. 1).

##### Remarks.

A diagnosis, based on the description of G1 is provided for *T.diabolis* since Pretzmann (1980) description and subsequent mentions of the species by other authors omitted important morphological details of G1. In *Tehuanadiabolis*G1 the proximal lobe of the caudo-marginal projection has an intermediate shape between those of *T.veracruzana* and *T.poglayenorum* (Fig. 8), and in the phylogenetic tree the three species appear also closely related (Fig. 3). Geographically, *T.diabolis* distribution around Lake Catemaco is also between that of *T.poglayenorum* to the northeast and that of *T.veracruzana* to the south of Catemaco Lake in the town of Zapoapan de Cabañas.

#### ﻿ Tehuana jacatepecensis

Taxon classificationAnimaliaAsteralesAsteraceae

Villalobos & Alvarez, 2003

2B98807D-2D6E-57FD-8A67-35F26EED5491

Figs 8H , 9H

Tehuana jacatepecensis Villalobos & Alvarez, 2003: 226, 228 (in key), figs 3, 4B.—Rodríguez and Magalhães 2005: 356, tab. 1 (list).—Villalobos Hiriart and Álvarez 2008: 280, 298 (list).—Ng et al. 2008: 177 (list).—Villalobos and Alvarez 2010: 475, 477, fig. 11 (map).—Mejía-Ortíz et al. 2011: 99, 136 (map 2).—Alvarez and Villalobos 2016: 254, tab. 8.1 (list).—Villalobos-Hiriart et al. 2019: 156, tab.1 (list).

##### Material examined.

Mexico – Oaxaca • 1 ♂, ***holotype***; CL 30.5 mm, CW 48.0 mm; Municipality of Santa María Jacatepec, Santo Domingo River in Santa María Jacatepec; 17°51'37"N, 96°12'36"W; alt. 54 m; 23 May 1992; L. Huidobro, C. Rosas, D. Becerril, R. Palma leg.; CNCR 11920. 2 ♂, 1 ♀, CL 11.9–25.9 mm, CW 17.6–39.9 mm; Municipality of San Juan Bautista Tuxtepec, km 165 highway Tuxtepec-Palomares, El Zapote stream; 17°09'51"N, 95°09'35"W; alt. 167 m; 27 Sep. 1981; R. Lamothe leg.; CNCR 8817. 3 ♂, 2 ♀, CL 15.9–24.4 mm, CW 24.1–37.2 mm; Municipality of Santa María Jacatepec, San Isidro El Naranjal, El Mazate waterfall; 17°53'41"N, 96°08'01"W; alt. 103 m; 3 Mar. 2018; J.L. Villalobos, I.A. Toledano, E. Moreno leg.; CNCR 34620. 5 ♂, 3 ♀, CL 11.4–28.1 mm, CW 16.6–43.7 mm; Municipality of Santa María Jacatepec, stream in San Isidro El Naranjal; 17°53'32"N, 96°07'46"W; alt. 84 m; 3 Mar. 2018; J.L. Villalobos, I.A. Toledano, E. Moreno leg.; CNCR 34622. 3 ♂, 7 ♀, CL 7.4–11.9 mm, CW 10–17 mm; Municipality of San José Chiltepec, Arroyo de Pueblo Viejo; 17°54'26"N, 96°03'12"W; alt. 79 m; 3 Mar. 2018; J.L. Villalobos, I.A. Toledano, E. Moreno leg.; CNCR 34640. 1 ♂, 1 ♀, CL 16.7–35.8 mm, CW 24.6–43.3 mm; Municipality of San Juan Bautista Tuxtepec, Tuxtepec-Palomares highway; 17°09'00"N, 95°06'00"W; alt. 96 m; collection data unknown; CNCR 8806. Veracruz • 2 ♂, 3 ♀, CL 9–43 mm, CW 12.8–67.3 mm; Municipality of Playa Vicente, Nueva Era; 1 km from Santa Rosa; 17°41'22"N, 95°48'55"W; alt. 111 m; 7 Mar. 2018; J.L. Villalobos, I.A. Toledano, E. Moreno leg.; CNCR 34624. 3 ♂, 3 ♀, CL 11.3–21.6 mm, CW 15.6–31.2 mm; Municipality of Playa Vicente, El Tomate, El Manantial Ranch, Manzo River; 17°41'52.3"N, 95°51'51"W; alt. 43 m; 6 Mar. 2018; J.L. Villalobos, I.A. Toledano, E. Moreno leg.; CNCR 34626.

##### Diagnosis.

As in Villalobos and Alvarez (2003).

##### Distribution.

This species is distributed in and around the town of Santa María Jacatepec in northern Oaxaca, Mexico (Fig. 1).

##### Remarks.

**Figure 9.**
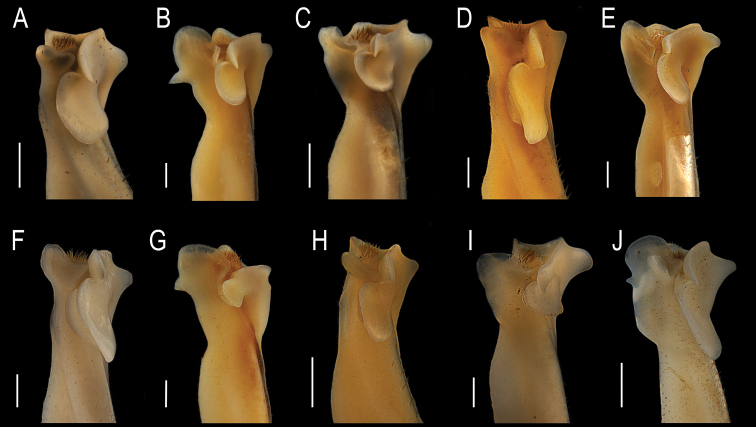
Cephalic view of the apical portion of the G1 of the species of *Tehuana***A***T.ayotzintepecensis* sp. nov., CNCR 34628 **B***T.chontalpaensis*, CNCR 17093 **C***T.col* sp. nov., CNCR 33928 **D***T.complanata*, CNCR 11957 **E***T.diabolis*, CNCR 12056 **F***T.lamellifrons*, CNCR 33939 **G***T.lamothei*, CNCR 8812 **H***T.jacatepecensis*, CNCR 11920 **I***T.poglayenorum*, CNCR 33931 **J***T.veracruzana*, CNCR 33934. Scale bars: 1 mm (**A, C, H, J**); 0.5 mm (**B, D, E, F, G, I**).

As noted by Villalobos and Alvarez (2003), the G1 of *T.jacatepecensis* is morphologically similar to those of *T.complanata* and *T.lamellifrons* (Figs 8, 9), and coincidentally the three species together with *T.ayotzintepecensis* sp. nov. are also closely related genetically forming a separate clade in the phylogenetic tree presented herein (Fig. 3).

#### ﻿ Tehuana lamellifrons

Taxon classificationAnimaliaAsteralesAsteraceae

(Rathbun, 1893)

AA97CE39-4291-581B-ADF3-6919C49918C4

Figs 8F , 9F

Pseudothelphusa lamellifrons Rathbun, 1893: 654, pl. 75, figs 2–5; 1898: 534, 537.— Young 1900: 221.—Rathbun 1905: 304.—Coiffman, 1939: 108.
Potamocarcinus lamellifrons .—Ortmann, 1897: 317 (key).
Pseudothelphusa (Pseudothelphusa) lamellifrons .—Pretzmann, 1965: 4.
Pseudothelphusa (Tehuana) lamellifrons lamellifrons .—Pretzmann, 1971: 22 (list).—Pretzmann 1972: 107, figs 612–614, 662–664.
Pseudothelphusa (Tehuana) lamellifrons .—Rodríguez & Smalley, 1972: 79, fig. 10.—Türkay 1978: 144.
Tehuana lamellifrons. —Rodriguez, 1982: 129, fig. 84.—Villalobos 1982: 220 (list).—Villalobos-Hiriart et al. 1993: 284 (list).—Alvarez and Villalobos 1994: 730.—Álvarez and Villalobos 1997a: 416, 417, fig. 4.17 (list).—Álvarez and Villalobos 1997b: 438, appx. 4.23 (list).—Álvarez et al. 1999: 20, fig. 3 (map).—Villalobos and Alvarez 2003: 228 (in key).—Villalobos Hiriart and Álvarez 2008: 282, 298 (list).—Ng et al. 2008: 177 (list).—Villalobos and Alvarez 2010: 475, 477, fig. 11 (map).—Guinot and Hendrickx 2014: 477.—Alvarez and Villalobos 2016: 254, tab. 8.1 (list).—Villalobos-Hiriart et al. 2019: 156, tab.1 (list).

##### Material examined.

Mexico – Oaxaca • 1 ♂, CL 50.4 mm, CW 80.6 mm; Municipality of Asunción Ixtaltepec, Nizanda, Cerro del Naranjo, Naranjo stream; 16°41'14.8"N, 95°02'09"W; alt. 293 m; 15 Apr. 1999; D. Barreto, V.H. Reynoso leg.; CNCR 16875. 1 ♂, CL 31.2 mm, CW 43.4 mm; Municipality of Asunción Ixtaltepec, Naranjo stream; 16°41'24"N, 95°22'53"W; alt. 639 m; 15 Sep. 1997; V.H. Reynoso leg.; CNCR 18951. 3 ♂, 2 ♀, CL 8.9–40.8 mm, CW 12.4–65 mm; Municipality of Asunción Ixtaltepec, Nizanda, stream; 16°39'30"N, 95°00'37"W; alt. 186 m; 26 Apr. 2017; J.L. Villalobos, I.A. Toledano, E. Moreno leg.; CNCR 33939.

##### Diagnosis.

G1 slender, almost straight. Meso-distal lobe conical, in mesial view somewhat compressed caudo-cephalically. Mesial process in transversal position relative to G1 main axis, laying over proximal lobe of caudo-marginal projection, reduced, rounded, superior margin projected distally, without lateral tooth; in cephalic view internal angle triangular, pointing towards distal lobe of caudo-marginal projection (Figs 8F, 9F). Lobes of CMP separated by long incision, lobes not gaping; in cephalic view proximal lobe oblique relative to main axis of gonopod, distal lobe slightly curved laterally. Distal lobe of caudo-marginal projection simple, rounded, projected cephalically; proximal lobe broadly rounded extending proximally, with internal semicircular carina. Apical cavity with elongated field of setae next to lateral margin; opening of spermatic channel in caudal position.

##### Type material.

The syntypes are deposited in the National Museum of Natural History, Smithsonian Institution, Washington, D.C. (USNM 3289).

##### Type locality.

Mexico, Oaxaca, Municipality of Santo Domingo Tehuantepec, Tehuantepec, Tehuantepec River; 16°18'60"N, 95°13'60"W; alt. 60 m (Rathbun, 1893).

##### Distribution.

Along the Pacific versant of the Isthmus of Tehuantepec, in the drainage systems of the Tehuantepec and Zanatepec rivers, in SW Oaxaca, Mexico (Fig. 1).

##### Remarks.

A new diagnosis for *T.lamellifrons* is here presented since those of Rathbun (1893) and Rodríguez and Smalley (1972) are too short omitting important characters of the G1. Other remarks see those for *T.jacatepecensis*.

#### ﻿ Tehuana lamothei

Taxon classificationAnimaliaAsteralesAsteraceae

Alvarez & Villalobos, 1994

628E2EB7-47D1-50F4-AF7A-D5300C15BB6B

Figs 8G , 9G

Tehuana lamothei Alvarez & Villalobos, 1994: 732, figs 2, 4c.—Álvarez and Villalobos 1995: 93.—Villalobos and Alvarez 2003: 228 (in key).—Rodríguez and Magalhães 2005: 356, tab. 1 (list).—Villalobos Hiriart and Álvarez 2008: 282, 298 (list).—Ng et al. 2008: 177 (list).—Villalobos and Alvarez 2010: 474, 477, fig. 11 (map).—Álvarez et al. 2011b: 289.—Cumberlidge et al. 2014: 147, tab. 7 (list).—Alvarez and Villalobos 2016: 254, tab. 8.1 (list).—Villalobos-Hiriart et al. 2019: 156, tab.1 (list).

##### Material examined.

Mexico – Chiapas • 1 ♂, ***holotype***; CL 18.2 mm, CW 27.5 mm; Municipality of Ixtacomitán, 1 km from Ixtacomitán, La Piedra stream; 17°24'00"N, 93°06'00"W; alt. 232 m; 4 Apr. 1986; J.L. Villalobos, J.C. Nates, A. Cantú, D. Valle; CNCR 5604. 2 ♂, CL 21–24 mm, CW 32–37.2 mm; Municipality of Tapilula, stream near Tapilula; 17°16'05"N, 93°01'33"W; alt. 780 m; 20 Apr. 1981; R. Lamothe leg.; CNCR 8812.

##### Diagnosis.

As in Alvarez and Villalobos (1994).

##### Distribution.

Restricted to a small area in NE Chiapas, Mexico (Fig. 1).

##### Remarks.

As in *T.chontalpaensis*.

#### ﻿ Tehuana poglayenorum

Taxon classificationAnimaliaAsteralesAsteraceae

(Pretzmann, 1978)

81534E80-9A36-5D9C-9215-455BE19F736A

Figs 8I , 9I

Pseudothelphusa (Tehuana) lamellifrons poglayenorum Pretzmann, 1978: 3; 1980: 660, pl. 12, figs 51–55.
Tehuana poglayenorum .—Villalobos-Hiriart et al. 1993: 284 (list).—Álvarez and Villalobos 1997a: 416, 417, box 4.17 (list); 1997b: 338, appx. 4.23 (list).—Álvarez et al. 1999: 20, fig. 3 (map), 23, box 3 (list).—Villalobos and Alvarez 2003: 228 (in key).—Villalobos Hiriart and Álvarez 2008: 283, 298 (list).—Villalobos and Alvarez 2010: 475, 477, fig. 11 (map).—Álvarez et al. 2011a: appx. VIII.20, p. 13 (list); 2012: 1078, box 1 (list).—Cumberlidge et al. 2014: 147, tab. 7 (list).—Alvarez and Villalobos 2016: 254, tab. 8.1 (list).—Villalobos-Hiriart et al. 2019: 156, tab.1 (list).
Tehuana poglayenora .—Ng et al. 2008: 177 (list) [error].
Tehuana lamellifrons .—Poettinger et al. 2016: 1722, tab. I (list).

##### Material examined.

Mexico – Veracruz • 4 ♂, CL 11.4–26.0 mm, CW 17.0–41.2 mm; Municipality of San Andrés Tuxtla, Basura River; 18°31'55"N, 95°03'30"W; alt. 33 m; 19 Jul. 1998; R. Robles, C. Graham leg.; CNCR 17422. 1 ♀, CL 15.8 mm, CW 22.9 mm; same locality as previous record; 18 Jul. 1986; J.L. Villalobos, F. Álvarez leg.; CNCR 13140. 3 ♂, 5 ♀, CL 8.9–28.8 mm, CW 13.1–45.2 mm; same locality as previous record; 4 Oct. 1994; J.L. Villalobos, F. Álvarez leg.; CNCR 13187. 7 ♂, 1 ♀, CL 9.6–21.8 mm, CW 14.7–36.4 mm; same locality as previous record; 24 Apr. 2017; J.L. Villalobos, I.A. Toledano, E. Moreno leg.; CNCR 33931. 4 ♂, 11 ♀, CL 11.8–19.5 mm, CW 12.8–33.2 mm; Municipality of Santiago Tuxtla, Tapalapan River; 18°32'00"N, 95°18'00"W; alt. 393 m; 17 Apr. 1957; H. Hobbs, A. Villalobos leg.; CNCR 336. 5 ♀, CL 25.1–36.1 mm, CW 40.6–59.1 mm; same locality as previous record; 23 May 1955; A. Villalobos leg.; CNCR 338. 4 ♂, 4 ♀, CL 7.3–14 mm, CW 10.8–20.3 mm; same locality as previous record; 17 April 1957; H. Hobbs, A. Villalobos leg.; CNCR 386. 2 ♂, CL 10.9–11.9 mm, CW 12.4–18.8 mm; Municipality of San Andrés Tuxtla, Otapan River; 18°26'00"N, 95°12'00"W; alt. 379 m; 22 Sep. 1955; G. Pérez leg.; CNCR 337. 2 ♂, CL 11.9–20.7 mm, CW 21.9–30.3 mm; Municipality of San Andrés Tuxtla, Laguna Escondida; 18°35'00"N, 95°05'00"W; alt. 76 m; 1 Aug. 1985; J.L. Villalobos, M.D. Valle, P. Schmidtsdorf leg.; CNCR 4473. 15 ♂, 10 ♀, CL 7.4–21.7 mm, CW 10.4–34.9 mm; same locality as previous record 13 Jun. 1985; C. Nates, J.L. Villalobos leg.; CNCR 4709. 1 ♂, 3 ♀, CL 8.2–25.6 mm, CW 11.6–41 mm; same locality as previous record; 11 Jul. 1994; J.L. Villalobos, F. Álvarez leg.; CNCR 5303. 4 ♂, 3 ♀, CL 25.4–32.3 mm, CW 42.2–53.2 mm; same locality as previous record; 24 Feb. 1989; M. Santiago leg.; CNCR 10220. 2 ♂, 2 ♀, CL 11–19.4 mm, CW 15.5–30.5 mm; same locality as previous record; 17 Jul. 1985; J.L. Villalobos, F. Álvarez leg.; CNCR 12908. 6 ♂, 4 ♀, CL 11.7–18.7 mm, CW 17.7–45.9 mm; same locality as previous record; 5 Aug. 1994; J.L. Villalobos leg.; CNCR 12964. 2 ♂, 2 ♀, CL 6.8–11.3 mm, CW 9.3–17.0 mm; same locality as previous record; 10 Jul. 1986; F. Álvarez leg.; CNCR 13138. 1 ♂, CL 20.0 mm, CW 32.3 mm; Municipality of San Andrés Tuxtla, Playa Escondida; 18°35'00"N, 95°03'00"W; alt. 6 m; 28 Feb. 1986; A. Cantú leg.; CNCR 5782. 3 ♂, 5 ♀, CL 13.3–17.2 mm, CW 20.0–27.0 mm; same locality as previous record; 28 Feb. 1986; A. Cervantes, J. García, A. Cantú leg.; CNCR 5788. 1 ♂, 4 ♀, CL 9.2–20.9 mm, CW 13.1–32.1 mm; same locality as previous record; 28 Feb. 1986; R. Lamothe leg.; CNCR 8821. 6 ♂, 11 ♀, CL 6.3–20.8 mm, CW 9.1–32.8 mm; Municipality of San Andrés Tuxtla, trail to Laguna Escondida; 18°35'00"N, 95°04'00"W; alt. 108 m; 24 Feb. 1989; M. Santiago leg.; CNCR 10222. 1 ♂, 2 ♀, CL 11.8–21.7 mm, CW 17.3–35.1 mm; same locality as previous record; 3 Aug. 1994; F. Álvarez, J.L. Villalobos leg.; CNCR 12967. 9 ♂, 5 ♀, CL 9.7–28.6 mm, CW 14.3–49.7 mm; same locality as previous record; 23 Apr. 2017; J.L. Villalobos, I.A. Toledano, E. Moreno leg.; CNCR 33927. 13 ♂, 21 ♀, CL 9.9–30.0 mm, CW 14.7–50.4 mm; Municipality of San Andrés Tuxtla, Lázaro Cárdenas;18°34'00"N, 95°06'00"W; alt. 359 m; 9 Jul. 1991; A. Cruz leg.; CNCR 12302. 1 ♂, CL 20.3 mm, CW 32.3 mm; Municipality of San Andrés Tuxtla, Cuetzalapan River; 18°24'00"N, 95°00'00"W; alt. 220 m; 13 Jul. 1986; F. Álvarez leg.; CNCR 12555. 2 ♂, 1 ♀, CL 11.3–23.6 mm, CW 18.0–37.7 mm; Municipality of San Andrés Tuxtla, La Palma River; 18°33'00"N, 95°03'00"W; alt. 77 m; 12 Jul. 1986; F. Álvarez leg.; CNCR 12896. 1 ♂, 1 ♀, CL 21.1–23.0 mm, CW 35.0–38.5 mm; Balzapote River; 18°36'00"N, 95°04'00"W; alt. 54 m; Municipality of San Andrés Tuxtla, Veracruz; 14 Jul. 1986; F. Álvarez leg.; CNCR 12906. 7 ♂, 3 ♀, CL 13.0–20.3 mm, CW 20.0–32.5 mm; Municipality of San Andrés Tuxtla, Máquinas River; 18°37'00"N, 95°05'00"W; alt. 143 m; 1 Aug. 1994; F. Álvarez, J.L. Villalobos leg.; CNCR 12961. 1 ♂, 2 ♀, CL 10.1–18.3 mm, CW 16.1–21.6 mm; Municipality of San Andrés Tuxtla, Zacatal Lagoon; 18°35'00"N, 95°06'00"W; alt. 263 m; 14 Oct. 1994; F. Álvarez, J.L. Villalobos leg.; CNCR 13188. 4 ♂, CL 11.8–23.4 mm, CW 17.5–37.2 mm; Municipality of Santiago Tuxtla, Simapan River; 18°27'00"N, 95°21'00"W; alt. 336 m; 3 May 1995; J.L. Villalobos leg.; CNCR 13346.

##### Diagnosis.

G1 slender, in mesial view distal third inclined cephalically. Meso-distal lobe conical, tip rounded, large relative to size of other apical structures (Fig. 9I). Mesial process well developed; lateral margin with triangular, acute tooth; distal margin broadly rounded; internal angle with triangular projection in cephalic position. Lobes of the caudo-marginal projection elongated, overlapping along most of their length; proximal lobe curved cephalically, distal lobe straight, oriented forward or with the tip moderated curved proximally, slightly longer than proximal one. In mesial view, distal lobe with superior margin inclined proximally; distally, falling over proximal lobe. Internal carina of proximal lobe rounded, prominent, separated from lobe. Apical cavity with elongated field of setae, opening of spermatic channel in caudal position.

##### Type material.

The holotype (NHMW 4066) and paratypes (NHMW 4067) are deposited in the Natural History Museum in Vienna, Austria.

##### Type locality.

Mexico, Veracruz, Municipality of San Andrés Tuxtla, Basura River; 18°32'00"N, 95°03'00"W; alt. 33 m (Pretzmann, 1978).

##### Distribution.

*Tehuanapoglayenorum* is distributed in the north-central section, of the Los Tuxtlas region.

##### Remarks.

*Tehuanapoglayenorum* is the most widely distributed and abundant freshwater crab in the whole Los Tuxtlas Mountain Range. It belongs to clade c, where all the species from Los Tuxtlas are grouped (Fig. 1). We noted there small morphological variations through its distribution range: in the carapace with setae or without them, and in the G1 in the caudo-marginal projection and mesial process.

Poettinger et al. (2016) presented the sequence of the16S mitochondrial gene (KU578859) as belonging to *T.lamellifrons*; however, the two females in the lot NHMW 4067 (from Río Basura, state of Veracruz) are the paratypes of *T.poglayenorum* designated by Pretzman (1978, 1980). |

#### ﻿ Tehuana veracruzana

Taxon classificationAnimaliaAsteralesAsteraceae

(Rodríguez & Smalley in Smalley 1970)

E6C45F73-407E-58EB-982C-A120DF10A90C

Figs 8J , 9J

Pseudothelphusa (Tehuana) veracruzana Rodríguez & Smalley in Smalley, 1970: 100, fig. 11. Pretzmann 1972: 108.—Rodríguez and Smalley 1972: 77, fig. 9.—Türkay 1978: 148, fig. 4.—Guinot and Hendrickx 2014: 478, tab. 1 (list).
Tehuana veracruzana .—Rodriguez, 1982: 131, fig. 86.—Villalobos 1982: 221 (list).—Rodríguez 1986: 59, fig. 10l, 61, fig.12—Villalobos-Hiriart et al. 1993: 284.—Álvarez and Villalobos 1995: 93; 1997a: 416, 417, box 4.17 (list); 1997b: 338, appx. 4.23 (list).—Álvarez et al. 1999: 20, fig. 3 (map), 23, box 3 (list).—Villalobos and Álvarez 2003: 228 (in key).—Villalobos Hiriart and Álvarez 2008: 284, 298 (list).—Ng et al. 2008: 177 (list).—Villalobos and Alvarez 2010: 474, 477, fig. 11 (map).—Álvarez et al. 2011a: appx. VIII.20, p. 13 (list); 2012: 1078, box 1 (list).—Guinot and Hendrickx 2014: 477.—Cumberlidge et al. 2014: 147, tab. 7 (list).—Alvarez and Villalobos 2016: 254, tab. 8.1 (list).—Villalobos-Hiriart et al. 2019: 156, tab.1 (list).

##### Material examined.

Mexico – Veracruz • 1 ♂, ***holotype***; CL 48.0 mm, CW 28.2 mm; Municipality of Catemaco, Zapoapan de Cabañas Stream; 18°20'00"N, 95°05'48"W; alt. 518 m; 15 Apr. 1957; A. Villalobos, H.H. Hobbs leg.; CNCR 335. 1 ♂, 1 ♀, ***paratypes***; CL 23.8–28.2 mm, CW 37.2–47.5 mm; same data as for holotype; CNCR 335. 1 ♂, 1 ♀, CL 15.7–27.8 mm, CW 24.8–45.2 mm; Municipality of Catemaco, stream near Zapoapan de Cabañas; 18°20'09"N, 95°02'22"W; alt. 629 m; 25 Apr. 2017; J.L. Villalobos, I.A. Toledano, E. Moreno leg.; CNCR 33934. 1 ♂, 3 ♀, CL 12.1–20.5 mm, CW 18.4–32.5 mm; Municipality of Catemaco, stream near Zapoapan de Cabañas; 18°20'32"N, 95°04'13"W; alt. 364 m; 25 Apr. 2017; J.L. Villalobos, I.A. Toledano, E. Moreno leg.; CNCR 33932. 1 ♂, 2 ♀, CL 9.2–12.5 mm, CW 14.5–21.6 mm; Municipality of Catemaco, stream near Zapoapan de Cabañas; 18°19'18"N, 95°03'02"W; alt. 364 m; 25 Apr. 2017; J.L. Villalobos, I.A. Toledano, E. Moreno leg.; CNCR 33937. 1 ♂, 1 ♀, CL 9.8–20.5 mm, CW 14.4–32.2 mm; Municipality of Catemaco, road Zapoapan de Cabañas–Zoteapan; 18°17'52"N, 94°58'06"W; alt. 848 m; 25 Apr. 2017; J.L. Villalobos, I.A. Toledano, E. Moreno leg.; CNCR 33955.

##### Diagnosis.

As in Rodríguez and Smalley (1972).

##### Distribution.

This species occurs in the southeastern portion of the Los Tuxtlas Mountain Range, in an area that starts sloping towards the coastal plain of southern Veracruz.

##### Remarks.

*Tehuanaveracruzana* is easily distinguishable from the rest of its congeners due to the very large proximal lobe of the CMP of the G1 (Fig. 8). Although it exhibits an extreme form of the G1 it is genetically closely related to the other species from Los Tuxtlas (Fig. 1).

## ﻿Discussion

The ten species of the genus *Tehuana* are distributed in the Isthmus of Tehuantepec, from central Veracruz to northern Chiapas (Fig. 1). The Isthmus of Tehuantepec has a complex geologic history being the area where three tectonic plates interact: North American, Cocos, and Caribbean (Barrier et al. 1998). As a result, the region has an intricate geography that has deeply influenced the evolution of many biological groups (e.g., Mendonça et al. 2022). It has also acted as a barrier for dispersal, promoting speciation and lineage differentiation (Gutiérrez-García and Vázquez-Domínguez 2013). Many examples from mammals and birds to plants, insects, and amphibians (Ornelas et al. 2013; Mendoza et al. 2019), show that the region harbors a high diversity and several areas of endemism. Interestingly, the Isthmus has acted as a barrier at different times for different groups of organisms.

For pseudothelphusid crabs, the Isthmus of Tehuantepec is a region where 16 genera belonging to three subfamilies occur, representing the highest diversity of lineages in the whole range of the family (Villalobos and Álvarez 2010; Álvarez et al. 2020). The involved genera represent major lineages, one with Central American affinity (subfamily Potamocarcininae), a second one that encompasses southern Mexico and northern Central America (subfamily Raddausinae) and a third one from central and northwestern Mexico (subfamily Pseudothelphusinae) (Álvarez et al. 2020).

The genus *Tehuana* is distributed entirely within the Isthmus of Tehuantepec, forming three groups, as indicated in the phylogenetic tree (Fig. 1). A salient feature is that they occupy both the Gulf of Mexico and Pacific slopes. In contrast to other groups of species, especially in the genus *Pseudothelphusa*, where progressive variation of the G1 morphology can be seen, in *Tehuana* the morphological variation of the CMP and MP do not follow a geographic gradient. The phylogenetic analysis groups species that are not morphologically the most similar, one example is the clade formed by the species from Los Tuxtlas region which show significant variation in the CMP.

According to Álvarez et al. (2020)*Tehuana* is one of the most recent groups to appear within the subfamily Pseudothelphusinae; the age estimate presented by these authors (2.5–0.5 mya) explains the relative short branches obtained in our phylogenetic tree (Fig. 3). The branching pattern within *Tehuana* we obtained has a geographic correlation with clade a, being the oldest one in Tabasco and Chiapas, Clade b, occurring in a diagonal band the stretches from central Veracruz to Oaxaca, and clade c, restricted to Los Tuxtlas region in Veracruz (Fig. 3). This progression suggests that the species in the genus radiated from southwest to northeast within the Isthmus of Tehuantepec.

## Supplementary Material

XML Treatment for Tehuana

XML Treatment for Tehuana ayotzintepecensis

XML Treatment for Tehuana col

XML Treatment for Tehuana complanata

XML Treatment for Tehuana chontalpaensis

XML Treatment for Tehuana diabolis

XML Treatment for Tehuana jacatepecensis

XML Treatment for Tehuana lamellifrons

XML Treatment for Tehuana lamothei

XML Treatment for Tehuana poglayenorum

XML Treatment for Tehuana veracruzana
